# Rare copy number variants in over 100,000 European ancestry subjects reveal multiple disease associations

**DOI:** 10.1038/s41467-019-13624-1

**Published:** 2020-01-14

**Authors:** Yun Rose Li, Joseph T. Glessner, Bradley P. Coe, Jin Li, Maede Mohebnasab, Xiao Chang, John Connolly, Charlly Kao, Zhi Wei, Jonathan Bradfield, Cecilia Kim, Cuiping Hou, Munir Khan, Frank Mentch, Haijun Qiu, Marina Bakay, Christopher Cardinale, Maria Lemma, Debra Abrams, Andrew Bridglall-Jhingoor, Meckenzie Behr, Shanell Harrison, George Otieno, Alexandria Thomas, Fengxiang Wang, Rosetta Chiavacci, Lawrence Wu, Dexter Hadley, Elizabeth Goldmuntz, Josephine Elia, John Maris, Robert Grundmeier, Marcella Devoto, Brendan Keating, Michael March, Renata Pellagrino, Struan F. A. Grant, Patrick M. A. Sleiman, Mingyao Li, Evan E. Eichler, Hakon Hakonarson

**Affiliations:** 1 The Center for Applied Genomics, The Children’s Hospital of Philadelphia, Philadelphia, Pennsylvania 19104 USA; 2 Department of Pediatrics, University of Pennsylvania Perelman School of Medicine, Philadelphia, Pennsylvania 19104 USA; 3 Helen Diller Comprehensive Family Cancer Center and Department of Radiation Oncology, University of California San Francisco, San Francisco, California 94143 USA; 4 Department of Genome Sciences, University of Washington School of Medicine, Seattle, Washington 98195 USA; 5 Affiliated Cancer Hospital and Institute of Guangzhou Medical University, Guangzhou, China; 6 Department of Computer Science, New Jersey Institute of Technology, Newark, New Jersey 07102 USA; 7 Division of Cardiology, The Children’s Hospital of Philadelphia, Philadelphia, Pennsylvania 19104 USA; 8 Department of Psychiatry, University of Pennsylvania School of Medicine, Philadelphia, Pennsylvania 19104 USA; 9 Department of Child and Adolescent Psychiatry, The Children’s Hospital of Philadelphia, Philadelphia, Pennsylvania 19104 USA; 10 Division of Oncology, The Children’s Hospital of Philadelphia, Philadelphia, Pennsylvania 19104 USA; 11 Center for Biomedical Informatics, The Children’s Hospital of Philadelphia, Philadelphia, Pennsylvania 19104 USA; 12 Division of Genetics, The Children’s Hospital of Philadelphia, Philadelphia, Pennsylvania 19104 USA; 13 Dipartimento di Medicina Sperimentale, University La Sapienza, 00185 Rome, Italy; 14 Department of Biostatistics and Epidemiology, University of Pennsylvania School of Medicine, Philadelphia, Pennsylvania 19104 USA; 15 Howard Hughes Medical Institute, University of Washington School of Medicine, Seattle, Washington 98195 USA

**Keywords:** Genome-wide association studies, Structural variation, Genetic predisposition to disease, Diseases

## Abstract

Copy number variants (CNVs) are suggested to have a widespread impact on the human genome and phenotypes. To understand the role of CNVs across human diseases, we examine the CNV genomic landscape of 100,028 unrelated individuals of European ancestry, using SNP and CGH array datasets. We observe an average CNV burden of ~650 kb, identifying a total of 11,314 deletion, 5625 duplication, and 2746 homozygous deletion CNV regions (CNVRs). In all, 13.7% are unreported, 58.6% overlap with at least one gene, and 32.8% interrupt coding exons. These CNVRs are significantly more likely to overlap OMIM genes (2.94-fold), GWAS loci (1.52-fold), and non-coding RNAs (1.44-fold), compared with random distribution (*P* < 1 × 10^−3^). We uncover CNV associations with four major disease categories, including autoimmune, cardio-metabolic, oncologic, and neurological/psychiatric diseases, and identify several drug-repurposing opportunities. Our results demonstrate robust frequency definition for large-scale rare variant association studies, identify CNVs associated with major disease categories, and illustrate the pleiotropic impact of CNVs in human disease.

## Abstract (web summary)

Associations of copy number variations (CNVs) with complex traits are challenging to study because of their low frequency. Here, the authors analyse SNP array and array comparative genomic hybridization data of 100,028 individuals and report their associations with immune-related, cardiometabolic and neuropsychiatric diseases as well as cancer.

## Introduction

Copy number variants (CNVs) are losses or gains of genomic segments. Although CNVs are commonly observed in healthy individuals, they are known to have gene dosage-sensitive effects on specific phenotypes. Prior CNV studies suggest that CNVs have a widespread impact on the human genome, as they are often associated with biological functions impacting disease susceptibility. However, most existing studies are based on limited sample sizes and lack adequate depth of rare CNV coverage. Historically, rare large CNVs are known to be associated with certain rare disease phenotypes. More recently, many previously thought to be rare  CNVs were found to be common across populations with allelic properties similar to single-nucleotide polymorphism (SNP) genotypes. The frequency and distribution of CNVs across the human genome have been examined by a number of recent studies using a variety of oligonucleotide arrays, CNV calling algorithms, and analytical methods, in an effort to understand the role of CNVs in human health and disease^1–12^. As CNVs impacting gene products are often deleterious, most such CNVs are rare genomic events that undergo negative selection. Consequently, large-scale sample sizes are required for sufficiently powered studies.

This study examines the CNV burden in over 100k subjects of European ancestry. We highlight the importance of rare, recurrent CNVs on the functional human genome and show that they are associated with on common, complex human diseases, including unreported therapeutic opportunities. Copy number variation regions (CNVRs) are significantly more likely to overlap OMIM genes (2.94-fold), genome-wide association studies (GWAS) loci (1.52-fold), and non-coding RNAs (ncRNAs; 1.44-fold), compared with random distribution (*P* < 1 × 10^−3^). We uncover strong CNV associations with four major disease categories, including autoimmune, cardio-metabolic, oncologic, and neurological/psychiatric diseases, several of which impact genes that represent potential drug targets for future validation.

## Results

### Amassing and curating CNV calls

To examine the distribution of CNVs across the human genome, we genotyped 100,028 individuals from populations of European ancestry using results from either genome-wide SNP arrays (Illumina and Affymetrix) or array comparative genomic hybridization (aCGH) platforms (SignatureChip and Agilent). For the SNP array platforms (*N* = 52,321), CNVs were called using both the genotype B allele frequencies and intensity log R ratios (LRRs) calculated from 520,017 overlapping SNPs; LRRs were used for CNV calls on the aCGH platforms (*N* = 48,707), see Supplementary Methods. For quality control and association testing, we used ParseCNV, a robust CNV pipeline that has been validated for CNV calling based on extensive experimental experience^13–17^. To avoid study bias in the interpretation of the frequencies of rare recurrent CNVs across the 100,028 subjects, individuals with known multi-systemic syndromic disorders with established causality attributable to CNVs were excluded from the analysis (i.e., 22Q deletion and duplication syndromes, del15q11-13, del16p11.2, and trisomy 21. Incidentally, apart from trisomy 21, through our genetic analysis, we identified genetic lesions associated with other syndromic conditions from subjects who were previously undiagnosed).

**Fig. 1:**
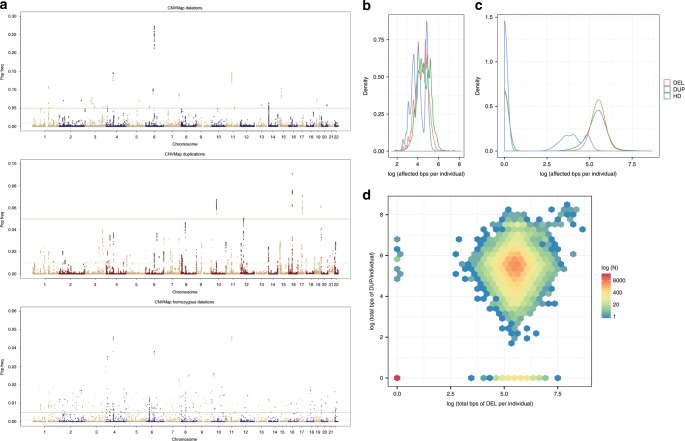
Genome-wide frequency and distribution of CNVs. **a** “Manhattan plot” showing the distribution of identified CNVs across the genome with genomic positions across the *x*-axis, including deletions (left), duplications (center), and homozygous deletions (bottom) with the *y*-axis showing observed frequency of each CNV in the total study population. **b** Histogram of duplication, deletion, and homozygous deletions frequencies for all identified CNVs as a function of total CNV burden per individual (in bps on a log10 scale). **c** Histogram of duplication, deletion, and homozygous deletions frequencies as a function of total CNV burden (in bps on a log10 scale) per individual. **d** Density plot showing each individual’s deletion burden (in bps on a log10 scale) plotted against that same individual’s duplication burden (in bps on a log10 scale). Individuals with the same event burden (bps affected) are binned into hexagon and the color correlates with the total count of individuals in each bin, reflecting that most individuals who have more duplications also have more deletions; however, there are some exceptions in patients with relatively large burdens of deletions or duplications without comparable burden of the converse.

We identified a total of 11,314 deletion, 5625 duplication, and 2746 homozygous-deletion CNVRs, defined as a contiguous cluster of non-singleton SNPs (occurring with a frequency of >0.03% or ~30 samples) that spans <1 Mb (Fig. 1a, Supplementary Fig 1, and Supplementary Data 1–3). We observed a mean CNV inheritance rate of 94% in a subset of several thousand family trios examined, with biological replicate concordance rate of 98%, confirming the reliability and specificity of the reported CNV calls (see Supplementary Methods). Furthermore, experimental validation by quantitative PCR (qPCR)^18^ was observed in >95% of the 2127 randomly selected, genotyped samples. We also examined CNVR distributions by genotyping platforms and CNVR size ranges to exclude the presence of technical bias in genotyping, sample processing, or CNV calling. As a further method of quality control, we showed that CNVRs observed across this study population recapitulated a number of CNVs previously reported by Conrad et al.^19^ (Supplementary Table 1).

A vast majority of CNVRs (11,287 deletions [99.75%] and 5614 [99.96%] duplications) were recurrent, or present in at least 2 individuals. Although individual CNVRs are rare (>98.5% had a frequency < 0.01), consistent with previous reports^20^, the presence of CNVs in the human genome is collectively common. Indeed, the average genome contained a total CNV burden of ~655 kb, of which 370 kb is accounted for by duplications, and 285 kb by deletions, and 9.5 kb of which are accounted for by homozygous deletions (Fig. 1b and Supplementary Fig. 2). Although CNVRs were heterogeneously distributed across the genome, no large stretch of the genome was exempt from harboring CNVs (Fig. 1a) and nearly all CNVRs (99.5%) harbor both deletions and duplications without a bias in mean deletion vs. duplication frequency (0.60% vs. 0.50%, respectively). Importantly, 13.7% of all CNVRs observed are unreported, defined as overlapping <50% with any CNVR reported in the Database of Genomic Variation (DGV).

### CNV impact on health and disease

Among the most clinically important CNVRs are homozygous deletions (hdCNVRs), as they are most likely to be pathologic. As expected, a lower percentage of the hdCNVRs are recurrent (2076 or 75.6%). Among the hdCNVRs identified, 375 are unreported, 44.3% of which were private. Leveraging the power of the large cohort, we also examined the data for evidence of homozygous or hemizygous deletions that incur embryonic lethality. Among the 2021 deletion CNVRs with a population frequency of at least 1.25%, we identified 62 loci at which no hdCNVR was identified, consistent with the possibility of embryonic lethality or early death (*P* < 0.05; Supplementary Table 2).

**Fig. 2:**
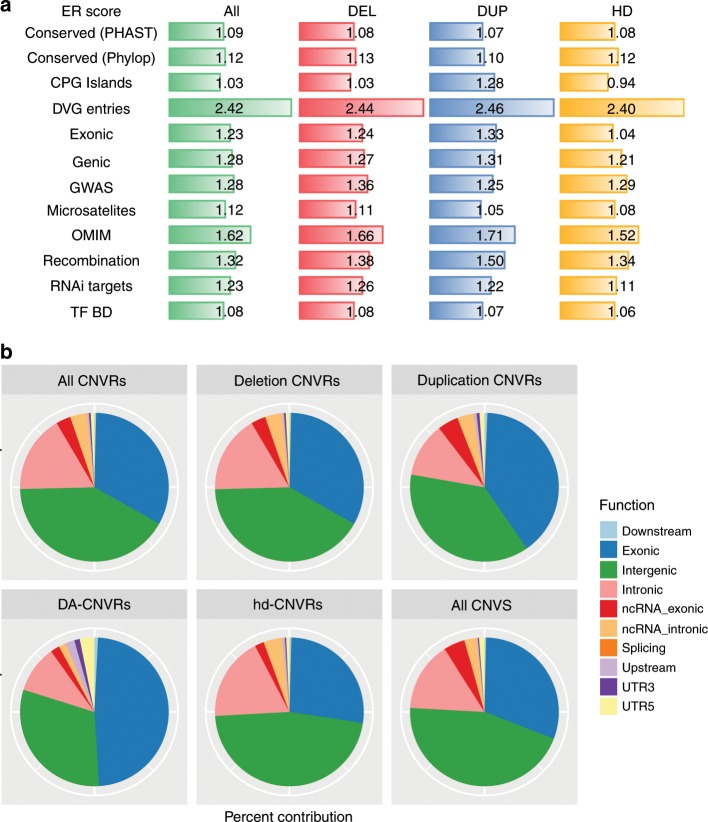
Functional enrichment analysis. **a** Relative enrichment ratios for mapping of a CNV to a locus annotated as each of the given categories. ER was calculated based on 10,000 repeated simulations for each category/CNVR combo (see Methods). Regions of the genome bearing CNVs were significantly more likely to map to loci that are gene-bearing (Genic regions), exonic, or conserved (PHAST or PhyloP), and are enriched for functional loci, including miRNA targets (RNAi), CpG islands (CpG), microsatellites, recombination hotspots (Recombination), transcription factor-binding sites (TF BD), and were more likely to map to regions previous CNVs have been reported in the Database for Genomic Variants (DGV). ER enrichment ratio; DUP duplication; DEL deletion; HD homozygous deletion. The enrichment results are reported separately for “ALL” CNVRs and CNVRs optimized for Dup, Del, or HD CNVs. **b** Contribution of CNVRs with different transcriptional impact as annotated by Ensemble Variant Effect Predictor for the nearest gene, respectively, for all CNVRs, deletion CNVRs, duplication CNVRs, disease-associated CNVRs, homozygous deletion CNVRs, and all CNVs identified.

To evaluate the biological and functional impact of CNVRs on human health and disease, we used repeated permutations to determine whether the overlap between CNVR regions and functional regions (RefSeq genes, OMIM morbid loci, ultra-conserved elements, CpG islands, conserved transcription factor-binding sites, ncRNAs, exons, conserved transcriptional factor-binding sites and genome-wide significant GWAS hits) was greater than that expected at random (see online methods; Fig. 2 and Supplementary Table 3). We identified significant annotations for both OMIM morbid genes (enrichment ratio, ER = 2.94), genome-wide significant GWAS loci (ER = 1.52), as well as recombination hotspots (ER = 1.32), all *P* *<* 1 × 10^−4^. Together, these findings underscore the important impact of natural selection in driving human genetic diversity and CNV distribution, as well as the importance of CNVs in phenotypic variation and human disease.

**Fig. 3:**
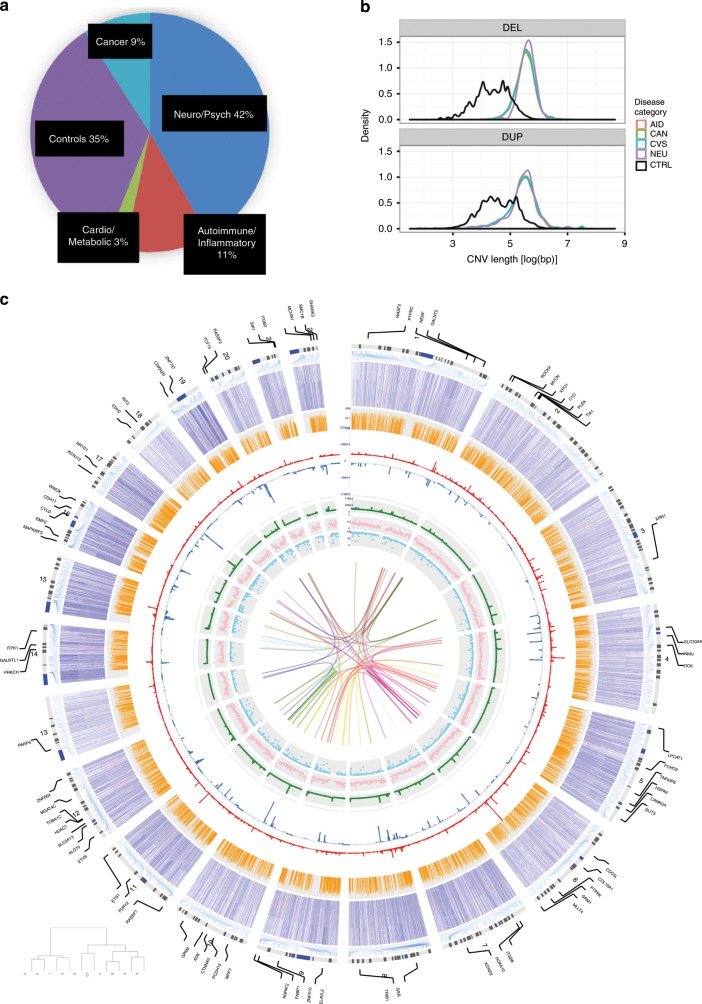
Genomic landscape of disease-associated CNVs. **a** Subjects (*n*) that are enrolled in the study based on disease category. Patients are classified into one of the four major disease categories or healthy controls. **b** Distribution of disease-associated deletions (top) and duplications (bottom) of CNVRs by length. Disease categories are color-coded. CNVRs associated with disease categories are on average larger than CNVRs not associated with disease categories (black-colored line), but do not significantly differ from one another. **c** Circos plot illustrating the distribution of CNVRs identified in the context of genomic elements. For all other layers from INNERMOST to OUTERMOST, the tracks show: sno/miRNAs (1), miRNA targets (2), conserved (*PHAST*) sites (3), frequency of duplication CNVRs (4), frequency of deletion CNVRs (5), recombination rates (*r*^*2*^) (6), expression of major EnCODE cell lines (7) corresponding to the candidate genes impacted by the respective DA-CNVRs (8). The innermost linkages reflect genes encoding the respective protein for all pairwise protein–protein interactions affected DA-CNVRs.

To identify potential associations between recurrent CNVs and human disease, we clustered individuals with disease phenotypes into four major categories (Fig. 3a) and healthy controls: (1) autoimmune/inflammatory diseases (*n* = 11,489), (2) cancers (*n* = 9105), (3) cardiovascular and metabolic diseases (*n* = 2581), and (4) a combination of psychiatric, neurodevelopmental, and neurological diseases (*n* = 43,841). At the expense of reduced sensitivity, this approach enabled us to identify loci that have broad disease implications in spite of the low frequency of most CNVRs, which makes a phenome-wide approach impractical^21^.

Consistent with expectation, disease-associated CNVRs (DA-CNVRs) are larger than average (Fig. 3b) and 94% (ER = 2.4) overlapped CNV-bearing regions previously reported in the DGV and 18% (ER = 1.2) overlapped GWAS loci (Fig. 2a), both significantly enriched as compared with chance (*P* < 0.001)^22,23^. We identified candidate genes in DA-CNVRs in a number of well-established disease-associated loci, including chr2p24.3 (*MYCN* amplification in cancer)^24^, chr22q11.21 (*COMT* and *TBX1* deletion in neuropsychiatric disease and congenital heart conditions)^25–27^, and chr17q21.1 (*NR1D1*, deletion and duplication associated with response to lithium in bipolar disease and major depressive disorder)^28,29^. In addition, we unveiled multiple, putatively unreported DA-CNVRs that map to relevant candidate genes (Table 1). Among those are several well-established drug targets and others in development, including but not limited to *TNFAIP8*, *HSPA9*, *SLIT3*, *HCN2*, *GRK6*, *ITGB8*, *ADK*, *CD44*, *NR1D1*, and *SLC38A10*^30–43^. We performed similar enrichment analyses across each set of the domain-specific DA-CNVRs, showing that a number of the functional and genomic elements were enrichments in multiple disease domains (Supplementary Fig. 5).

**Table 1:** Select loci enriched with CNVs in four major disease categories.

Pheno	Cyto band	Del/ Dup	*P*-value	Gene name	Annotations
Cardio	1p36.31	del	5.3E–14	*ACOT7*, *BACH*, *GPR153*	ACOT7 upregulation protects against fatty acid oversupply in the heart [21212523]
Neuro	1q21.1	del dup	6.0E–31 1.6E–27	*BC036212*, *KIAA1693*, *NBPF1*	*NBPF1* duplication in arteriovenous malformations [24098321]
Cancer	2p24.3	dup	2.4E–61	*MYCN*	Amplified/duplicated in neuroblastoma [23401364 and 15013217], DDX1-MYCN duplication in nephroblastoma [24161495]
Cancer	2q31.1	del	6.2E–26	*GPR155*, *SCRN3*	Overexpressed in breast cancer [19022662], deletion in osteosarcoma [15298715], CNV reduced hepatocellular carcinoma cell line expression [28863781]
Aid	4p13	del	1.7E–18	*SLC30A9*, *WDR21B*	Zn regulation in white blood cells [25927708]
Cancer	4p13	del	4.9E–23	*BEND4*, *CCDC4*, *SLC30A9*	BEND4 in colorectal cancer [21636702], CCDC4 in MDR pancreatic adenocarcinoma [18453221], deletion in bladder and other carcinomas [11906820]
Aid	4p16.3	dup	1.4E–21	*RNF212*	E3 Ubiquitin ligase, involved in meiotic recombination [23396135]
Neuro	4p16.3	both	2.5E–24	*TACC3*, *TMEM129*	Neurogenesis during cortical development [22842144], neuronal differentiation [20823227]
Neuro	5q23.1	dup	3.8E–27	*TNFAIP8*	Induced in Parkinson’s disease [24444419]
Cancer	5q31.2	del	3.5E–20	*HSPA9*	Medullary thyroid carcinoma [25435367], tumor suppressor signaling [23959801], oral SCC [23541579], HCC [17934217], colorectal cancer [15532096], brain tumors [9417864]
Neuro	5q35.1	del	3.9E–23	*SLIT3*	Parkinson’s disease [19162339]
Neuro	5q35.3	dup	6.6E–75	*GRK6*	Schizophrenia [21784156]
Neuro	6p22.1	dup	1.8E–52	*HIST1H2AE*, *HIST1H2BG*, *HIST1H4E*	HIST1H2BG associated with schizophrenia [23904455]
Cancer	7p12.1	dup	5.1E–58	*DKFZP564N2472*	Lung adenocarcinoma [21151896]
Cancer	7p12.2	del	3.7E–17	*IKZF1*, *ZNFN1A1*	Inactivation in lymphoma [11980663 and 11839096], deletion of IKZF1 or ZNFN1A1 in acute lymphoblastic leukemia [19129520, 26050650, 29519871, 15390181],
Aid	7p15.3	del	6.9E–19	*ITGB8*	Inflammatory bowel disease [28067908]
Cancer	8p22	del	7.5E–28	*SGCZ*	Mutated in AML [24189654], recurrent copy number loss in breast cancer [29545918], downregulation of MiR-383 in intron of SGCZ [28243881], gene fusions NCAM2-SGCZ in metastatic small-cell gallbladder neuroendocrine carcinoma [28040546]
Cancer	8q11.1	dup	2.0E–31	*AK097475*, *BC041354*	Upstream of *CMYC*, identified in prostate [16130124] and breast cancer [10867149, 15527903, 17213017]
Cancer	8q24.13	del	1.6E–17	*TRIB1*	Leukemia [27390356], double minute in acute myeloid leukemia [18503831], colon cancer [19691111], prostate cancer [24962028]
Neuro	10p14	dup	8.0E–19	*CUGBP2*, *NAPOR-1*	Neuroblastoma apoptosis-related RNA-binding protein [9858671]
Neuro	10q22.2	del	1.5E–23	*ADK*	Epilepsy and glioma [26329539]
Cancer	10q26.3	del	8.4E–15	*TCERG1L*	Silenced in colorectal cancer [22238052], higher methylation level in colorectal cancer [23546389, 22238052, 23321599]
Cancer	11p12	dup	2.7E–26	*C11orf74*, *near CD44*	Marker for Langerhans cell sarcoma [25837753], disregulation of CD44 in different types of cancer [25025570, 30631039, 30443182, 30211160, 30317669, 30463359]
Aid	11q13.4	del	1.8E–26	*UCP2*	Protective in sepsis [25873251], protective in inflammation [23925522], decrease the severity of multiple sclerosis in mice model [21857957]
Cancer	11q22.3	dup	9.0E–18	*SLC35F2*	NSCLC [21874247], an important molecular determinant of response to the Sepantronium Bromide [25064833, 28465296, 25568070]
Neuro	14q24.3	dup	5.9E–41	*C14orf43*	Pediatric pineal germinomas [27889662], vanishing white matter syndrome [22678813]
Aid	14q31.3	del	4.5E–23	*FLRT2*	Autoantigen in SLE [23401699], prostate cancer [26890304]
Cancer	16p13.3	del	4.4E–50	*MAPK8IP3*	Increased in brain tumor [16141199]
Neuro	16p13.3	del	1.7E–18	*SOX8*	Neural crest development [16943273], loss of Sox8 alleles in Hirschsprung disease [15572147]
Neuro	17q12	del	9.4E–18	*ACACA*, *C17orf78*	ACACA deleted in autism [23375656], ACACA associated with Alzheimer’s disease [22982105]
Neuro	17q21.1	dup	5.7E–73	*NR1D1*	Major depression [23671070], bipolar disorder [26746321, 25359533]
Aid	17q25.3	dup	6.1E–34	*SLC38A10*	Associated with N-glycosylation of human immunoglobulin G show pleiotropy with autoimmune diseases and haematological cancers [23382691]
Cancer	19p13.3	dup	7.6E–25	*PALM*	Cancer [28188128], CLL [28165464], lung cancer [25982285], near *STK11*, Peutz-Jehgers
Neuro	19p13.3	dup	6.5E–47	*HCN2*, *POLRMT*	Decreased HCN2 reduces learning abilities [21593326], HCN2 gene deletion decreased neuropathic pain [21903816]
Cardio	22q11.21	del	3.7E–23	*COMT*, *TBX1*	Velocardiofacial syndrome [26278718]

CNVRs presented in Table 1 show disease category-specific enrichment reaching statistical significance (*P* *<* 9 × 10^−14^), which is adjusted for multiple comparisons based on results obtained from repeated simulations (see Methods). See Supplementary Data 5 for extended results (*P* *<* 5 × 10^−8^). Brackets [notation] denote PMIDs

Finally, we also identified multiple disease-associated hdCNVRs (DA-hdCNVRs), including those that map to unreported disease-association loci, as well as those that map to known genes reported by GWAS or other association studies (Table 2). On average, DA-hdCNVRs are smaller in size than the DA-CNVRs, which may be a consequence of the deleteriousness of large hdCNVR events in the genome (Supplementary Fig. 5).

**Table 2:** Homozygous deletion CNVRs associated with a major disease category.

Pheno	Chr:Pos (hg18)	*P*-value	Cases	Controls	Gene name	Phenotype information
Cardio	chr11:55204003–55204003	8.0E–10	157	1204	*OR4C6*	Obesity [21131291]
Cancer	chr11:81194909–81194909	2.9E–07	80	129	*BC041900*	Target of FOXF2; deficiency important in E > M transition
Neuro	chr1:78432711–78432711	6.1E–06	20	6	*AX747165*, *BC015860*	Missense (c.785C > T; p.L262R) and nonsense (c.903G > A; p.W301X) mutations in human GIPC3 cause congenital sensorineural hearing impairment
Aid	chr1:167466049–167466049	3.0E–05	15	6	*NME7*	Venous thromboembolism
Neuro	chr13:97328242–97330758	3.8E–05	28	16	*IPO5*	Schizophrenia
Neuro	chr6:67105019–67105019	4.1E–05	97	110	*EYS*	AR retinitis pigmentosa
Cardio	chr5:113188389–113197319	4.2E–05	34	195	*YTHDC2*	mRNA metabolism
Aid	chr3:191217916–191217916	4.5E–05	28	23	*LEPREL1*	Homozygous loss-of-function mutation causes severe non-syndromic high myopia with early-onset cataracts.
Aid	chr12:27539678–27545813	8.1E–05	10	2	*PPFIBP1*	Receptor tyrosine kinase
Neuro	chr3:163625169–163625169	1.1E–04	57	55	*BC073807*	NA
Aid	chr5:117421055–117421055	2.5E–04	79	124	*BC044609*	NA
Neuro	chr19:40354649–40354649	2.6E–04	51	49	*FXYD5*	NA
Neuro	chr15:50050557–50057972	2.7E–04	12	3	*LEO1*	Neural tube development [20178782]
Aid	chr3:75511365–75532825	3.8E–04	57	82	*DQ584669*	N/A

CNVRs presented in Table 2 are select loci from those that reached experimentally defined statistical significance (*P* < 5 × 10^−4^), which adjusts for multiple comparisons based on results obtained from repeated simulations (see Methods). See Supplementary Data 6 for additional loci that are marginally associated with at least one disease category

## Discussion

Our analysis presents a dense map of CNVs across the human genome (Fig. 1 and Supplementary Data 1–3) and refines the frequencies of rare recurrent CNVs across the genome. Although individual CNVs are rare, most are recurrent and, collectively, CNVs represent an important and not infrequent source of genetic variation in the human genome. Although the importance of CNVs in rare genetic syndromes, congenital diseases, and in the cancer genome is well-elucidated, the role of germline CNVs in common human diseases has been thus far largely understudied. The salient overlap between CNVRs and GWAS signals observed in our study suggests that rare and uncommon CNVs may significantly contribute to common polygenic diseases (Fig. 2). Although some association studies have attempted to leverage GWAS to capture common CNVs, in the form of TagSNPs, our findings suggest that common polymorphisms do not effectively capture rare CNVs (Supplementary Data 4). This may  contribute to the missing heritability observed when comparing heritability calculated from GWAS findings with the expected heritability obtained from familial/twin studies. More effort is needed to evaluate the degree to which rare CNVs may contribute to the observed missing heritability using larger sample sizes and appropriate genomic platforms.

The role of CNVs and structural rearrangements as a driving force in human evolution and genome variation is also evident in the overlap of CNVRs with recombination hotspots. Recombination rates were higher for smaller and less common CNVRs and, in keeping with prior reports, a third (35.3%) of the CNVRs overlapped a recombination hotspot. In addition, we observed significant overlap between CNVRs and loci bearing segmental duplications and microsatellite sites (*P* < 1 × 10^−4^; Fig. 2a and Supplementary Figs. 3 and 4), likely related to increased rates of DNA replication defects at these regions. As polymorphic and repetitive loci are often neglected in disease-association studies due to technical challenges, it is especially important to further evaluate the phenotypic contributions of CNVs mapping to these loci.

We report herein a number of CNVRs, including a number of unreported CNVRs, which are associated with common human diseases. In addition to providing evidence of the impact of CNVRs in common diseases, these genes offer important avenues for therapeutic intervention. One notable example is a chr7p15.3 deletion associated with autoimmune disease (*P* < 6.87 × 10^−19^). This 2.9 kb deletion overlaps the gene *ITGB8*, which encodes the cell-surface glycoprotein β8 integrinITGB8a is a  well-established drug target for ovarian cancer and its expression is critical for dendritic cell-mediated induction of regulatory T-cell repertoires^44^. Through extensive functional studies, Travis et al.^45^ and others have shown that the conditional loss of the transforming growth factor-β-activating integrin α-V/β8 on leukocytes causes severe inflammatory bowel disease and age-related autoimmunity in mice. Recent efforts show that the α-V/β8 receptor complex is a viable therapeutic target in fibro-inflammatory airway disease^46^.

Another candidate locus is chr19p13.3, which encodes *HCN2*, a hyperpolarization-activated, cyclic nucleotide-gated K+ channel^47^. CNVs at this locus were associated with neurological diseases (deletion *P* < 6.53 × 10^−47^ and duplication *P* < 1.75 × 10^−11^). A careful review of literature reveals that increased *HCN2* expression and activity are associated with neuropathic hyperalgesia, and neuropathic pain is initiated by HCN2-driven action potential firing in NaV1.8-expressing nociceptors^48^. In addition, Dibbens et al.^49^ showed that a 9 bp exonic deletion in *HCN2* was associated with pediatric-onset generalized epilepsy with febrile seizures, consistent with the enhanced neuronal excitability observed in vitro in a *Xenopus* oocyte model of the indel. Several other genes residing at the association loci reported include known drug targets with repurposing opportunities, such as GRK6, CD44, SLC38A10, and ADK, with potential implications across multiple different cancers and auto-inflammatory diseases.

Moreover, in this work, we identified a number of DA-hdCNVRs. A prime example is an hdCNVR at chr2q34 that is associated with autoimmunity and interrupts the coding region of *ERBB4.* This gene encodes a cell-surface receptor tyrosine kinase that is a key oncogene and targetable by multiple Food and Drug Administration-approved small-molecule inhibitors. Interestingly, germline mutations in *ERBB4* have also recently been linked to amyotrophic lateral sclerosis and schizophrenia^50,51^, and the loss of ErbB4 expression is found in patients with relapsing–remitting multiple sclerosis^52^. Furthermore, the expression of this family of proteins in microglia promotes re-myelination of neurons in response to soluble isoform of neuregulin-1, also known as glial growth factor 2^53,54^. Given the safety and efficacy of established small-molecule modulators of this gene, it would be an ideal candidate gene for multi-disease, drug-repositioning opportunities.

Collectively, our findings support  a biological model in which recurrent CNVRs play a role across multiple common human diseases due to pleiotropic functions and/or broad expression of the affected gene(s). In support of this hypothesis, we examined the expression patterns of candidate genes in autoimmune/inflammatory-associated CNVRs and showed that their expression is enriched in immune-specific tissues and cell types, as compared with other genes in the human transcriptome (Supplementary Fig. 6b). In addition, we show extensive literature support of candidate genes in cancer-associated CNVRs and the pleiotropic expression patterns of these genes across multiple tissues and at high levels in malignant cells from these sites (Supplementary Fig. 8 and Supplementary Data 7). Finally, for CNVRs associated with multiple disease categories, we show that the impacted candidate genes are significantly more likely to be found in shared biological networks and to impact gene network interactions (Supplementary Fig. 7).

In summary, we report the frequencies, distributions, and disease associations of rare recurrent CNVs using genome-wide SNP genotyping and CGH data from over 100,000 unrelated individuals of European ancestry, representing the largest population-based CNV analysis to-date. The strong correlation between CNVRs and known GWAS loci, as well as recombination hotspots, suggest that rare or uncommon CNVs may contribute to the missing heritability conundrum, and that CNV-bearing loci correlate with regions of the genome under increased selective pressures. We further support this by demonstrating that many candidate genes mapping to DA-CNVRs are broadly expressed across multiple tissues and show examples in which such candidate genes have known pleiotropic roles across related diseases. Finally, although GWAS and genome-wide CNV analyses have significantly advanced our understanding of the distribution and biological impact of CNVs, whole-genome sequencing studies are needed to identify and fine-map CNVs, particularly those that are extremely rare and/or under 1 kb^55^. Together, our observations underscore that CNVs—both common and rare—have important biological roles in human health and disease. Thus, more effort and larger studies elucidating these associations may be an avenue for understanding and diagnosing genetic diseases and targeted therapeutic interventions.

## Methods

### Sample cohorts

All samples in the primary analysis were derived from one of two cohorts. The first group included 52,321 samples obtained from de-identified samples associated with electronic medical records residing in the genomics biorepository at The Children’s Hospital of Philadelphia (CHOP). All samples were also genotyped at the Center for Applied Genomics (CAG) at CHOP. A second set of 29,085 samples from subjects with neurological disease and 19,584 matching controls were run on a CGH array at The University of Washington in Seattle, WA. Informed consent authorizing the use of de-identified GWAS data was obtained from all subjects. The Institutional Review Board at The Children’s Hospital of Philadelphia approved the study.

Over 95% of the DNA was extracted from fresh blood. Six incremental versions of the Illumina 550k SNP set were used across both sets of samples, with a total of 520,017 SNPs in common to all included chip versions. PennCNV^56^ was used for CNV calls and validated by QuantiSNP^18^. To ensure data quality and to minimize technical bias, only samples with a mean call rate >98% and LRR SD < 0.25 were included in the analysis. Furthermore, autosome genotype relatedness and intensity wave variations following GC content wave correction were assessed for sample exclusion. CNV sensitivity was assessed based on the identification of known CNVs in the reference HapMap individuals. CNV calls of different size ranges across the genome were validated by independent testing in 2127 samples using qPCR with an overall specificity exceeding 95% as shown by results from 367 successful validation assays including consistent data quality across 7 disease studies with a range of different genomic loci and CN states (Supplementary Fig. 9).

### Quality filtering

To minimize false-positive CNV calls, a set of quality-filtering metrics were performed. Case and control matching was insured by calculating a genomic inflation factor between groups. Wave artifacts roughly correlating with GC content resulting from hybridization bias of low full-length DNA quantity are known to interfere with accurate detection of copy number variations. Only samples with GC-corrected wave factor of LRR < |0.02| were accepted. If the count of CNV calls made by PennCNV exceeded 100, it was suggestive of poor DNA quality and those samples were excluded. Thus, only samples with CNV call count <100 were included. Any duplicate samples (such as monozygotic twins or repeats on the same patient) were identified, and, as a result, one sample was excluded.

### Statistical tests

CNV frequency was compared between cases and controls, as well as across genotyping cohorts. Comparisons were made for each SNP using Fisher’s exact test. To determine CNV enrichment, we only considered loci that were nominally significant between the comparative groups (*P* *<* 0.05). For case–control comparisons, we looked for recurrent CNVs that were observed across different independent cohorts or were not observed in any of the control subjects, and were validated with an independent method. Three lines of evidence established statistical significance: independent replication *P* *<* 0.05, permutation of observations, and absence of loci observed with control enriched significance. We used DAVID (Database for Annotation, Visualization, and Integrated Discovery)^57^ to assess the significance of functional annotation clustering of independently associated results into InterPro categories.

We have complied with all relevant ethical regulations for the work with human participants. Informed consent was obtained for all participants prior to including them in the biobank at the CAG at CHOP.

## Supplementary information

Supplementary Information

Supplementary Dataset 1

Supplementary Dataset 2

Supplementary Dataset 3

Supplementary Dataset 4

Supplementary Dataset 5

Supplementary Dataset 6

Supplementary Dataset 7

Description of Additional Supplementary Files

## Data Availability

CNV calls generated are available from the authors and are published with open access on the website (https://www.filehosting.org/file/details/832375/Li_etal_NatureCommunications_SigConCag_hg18_rawcnv_bed.zip). All data authorized for dbGaP submission have been deposited to dbGaP (accessions: phs000490.v1.p1, phs000607.v3.p2, phs000371.v1.p1, phs000490.v1.p1, phs001194.v2.p2, phs001194.v2.p2.c1, phs001661, and phs000233).
