# Supplementary material for: Rare copy number variants in over 100,000 European ancestry subjects reveal multiple disease associations
Source: Nat Commun. 2020 Jan 14;11:255. doi: 10.1038/s41467-019-13624-1 (PMC6959272; doi:10.1038/s41467-019-13624-1)
Supplement: Supplementary file 1 — Supplementary Information [file 41467_2019_13624_MOESM1_ESM.docx]

**Supplementary Information**

Yun Rose Li^1,2,3#^, Joseph T. Glessner^1,2#^, Bradley P. Coe^4^, Jin Li^1,5^, Maede Mohebnasab^1^, Xiao Chang^1^, John Connolly^1^, Charlly Kao^1^, Zhi Wei^6^, Jonathan Bradfield^1^, Cecilia Kim^1^, Cuiping Hou^1^, Munir Khan^1^, Frank Mentch^1^, Haijun Qiu^1^, Marina Bakay^1^, Christopher Cardinale^1^, Maria Lemma^1^, Debra Abrams^1^, Andrew Bridglall-Jhingoor^1^, Meckenzie Behr^1^, Shanell Harrison^1^, George Otieno^1^, Alexandria Thomas^1^, Fengxiang Wang^1^, Rosetta Chiavacci^1^, Lawrence Wu^1^,  Dexter Hadley^3^, Elizabeth Goldmuntz^2,7^, Josephine Elia^2,8,9^, John Maris^2,10^, Robert Grundmeier^11^, Marcella Devoto^2,12,13,14^, Brendan Keating^1^, Michael March^1^, Renata Pellagrino^1^, Struan F.A. Grant^1,2^, Patrick M.A. Sleiman^1^, Mingyao Li^14^, Evan E. Eichler^15,16^, Hakon Hakonarson^1,2*^

**Supplementary Methods**

**DNA Samples, Array Platform and QC-Algorithms Used**

Both disease-free subjects and subjects with complex medical disorders were included in the study. We have categorized the phenotype datasets into five categories, representing: four major disease categories including autoimmune/inflammatory disease (n=11,489), cancer (n=9,105), cardiovascular and metabolic disease (n=2,581), neuro-psychiatric (including developmental delay and psychiatric disease (n= 29,085), and neurological disorders (n=14,756), in addition to healthy controls. All samples were typed at the CAG center within a five-year interval from August 2006 to July 2011. 29,085 neurological and 19,584 controls were further genotyped elsewhere. The majority of samples (over 95%) were extracted from fresh blood and genotyped using standard operating procedures (SOPs) developed by CAG.^1^ All data were tracked using a laboratory information management system (LIMS) developed by CAG and Illumina. DNA derived from cell lines have been shown to have increase in certain CNV artifacts.^2^ Since relatively few samples had cell line DNA source this was readily controlled for and all CNV loci reported included contributions from blood-derived DNA ruling out contributions from cell line artifacts.

The Illumina arrays used for genotyping included six incremental versions of the 550k SNP set. In, total 520,017 SNPs were common to all the chip versions and were defined as the intersection set that was subsequently assessed, using PennCNV^3^, with the PennCNV output subsequently validated by QuantiSNP.^4^ This was implemented by filtering the population frequency of the B allele (PFB) file. The differences between the Illumina platform chip versions was mitigated by evaluating only the intersection SNP set. The genotype and intensity data were evaluated in tandem to boost confidence in CNV calls. The high minor allele frequency SNPs are preferable for detection of CNVs. Assuming the diploid state of an individual is AB, a deletion would result in an A state and a duplication would result in a AAB or ABB state with different characteristics. The homozygote state does not differentiate when AA is in the CNV range since A and AAA both have the same pattern and only differ by count. The intensity data complement the genotype data by dimming regions of deletion and brightening regions of duplication.

PennCNV was used as the primary analysis tool to call CNVs in zero and one copy deletion states as well as three and four copy duplication states. A hidden Markov model was trained based on representative sample subgroups from our dataset. Based on population allele frequency, the SNP spacing, BAF and LRR, the maximum likelihood CNV state is determined for a contiguous set of SNPs. Technical replicates of multiple samples that were run two or more times on the Illumina array yielded high reproducibility. Family members were evaluated for inheritance of CNVs for further assurance of true positive CNV detection. We additionally applied QuantiSNP on the entire dataset for further validation of the CNV calls.

Quality metrics were calculated and their distributions assessed to ensure optimal quality and to minimize bias. Only samples with call rate >98% and Log R Ratio (LRR) standard deviation <0.35 were included in the analysis, reducing the need for experimental validation of the CNV calls made. Furthermore, autosome genotype relatedness, excessive CNV calls as a measure of poor sample quality, and intensity wave variations following GC content wave correction, were assessed for sample exclusion.^5,6^

CNV sensitivity was excellent, based on our detection rate of known CNVs in reference HapMap individuals and CNVs reported in the Database of Genomic Variants. CNV specificity was similarly found to be high, given positive independent experimental validation in 91% of 2,127 samples, testing different size ranges across the entire genome, using qPCR (**Sup. Fig. 9**). We validated both the presence and absence of CNV in various loci across randomly chosen samples. Furthermore, inheritance rate of CNVs was 94% and concordance between biological replicates was 100%.

When evaluated at the whole-genome level, CNVs show distribution in a heterogeneous manner throughout the genome and no large stretches of the genome are exempt from CNVs, with the proportion of any given chromosome susceptible to CNV varying from 46.7% to 96.1% (**Sup. Fig. 2),** due in part to SNP resolution.

**Additional Microarray platforms and samples**

We combined the 15,767 cases previously published in Cooper *et al*.^46^ with 13,318 new cases with intellectual disability and/or developmental delay and related phenotypes that were submitted to Signature Genomics Laboratories, LLC, for clinical microarray-based CGH. Array CGH was performed on nine different CGH platforms. All arrays were reanalyzed from the underlying raw data for CNVs. The majority of samples were profiled on an array with 135,000 or more probes (64%) with increased density in regions associated with known disorders^46,47^. Initial CNV calls were generated as previously described^47^.

We constructed a CNV atlas map by combining 8,329 controls from Cooper et al.^46^ (dbVar study accession nsdt54) with 11,255 new controls profiled on Affymetrix SNP6 arrays from the Wellcome Trust Case Control Consortium 2 (WTCCC2) 58C cohort, as well as the Atherosclerosis Risk in Communities (ARIC) Community Surveillance Cohort (database of Genotypes and Phenotypes (dbGaP) accession phs000090.v1.p1). All CNV calling for the ARIC and WTCCC2 58C cohorts was performed using GTC4.1 with default parameters, except for the minimum CNV size and minimum number of probes, which were set to 10 and 20 kb, respectively. Additional details on the 29,085 neurological and 19,584 controls cohort may be found in Coe *et al*^45^. With regard to overlapping samples, the intensity and genotype data from these array data were merged to provide more rigorous intra-sample quality control. This allowed for better evaluation for possible batch effects or systematic technical biases between runs and platforms. Any overlapping samples were subsequently removed (i.e., all samples reported on are unique samples).

**Illumina Infinium assay for SNP Genotyping and CNV Discovery**

The genotype data content together with the intensity data derived from the Infinium BeadChip technology (Illumina San Diego CA) as described above provides high confidence for CNV calls. Importantly, the simultaneous analysis of intensity data and genotype data in the same experimental setting establishes a highly accurate definition for normal diploid states and any deviation from this. To call CNVs, we used the PennCNV algorithm^3^, which combines multiple sources of information, including Log R Ratio (LRR) and B Allele Frequency (BAF) at each SNP marker, along with SNP spacing and population frequency of the B allele to generate CNV calls. Rare recurrent CNVs were the focus of our study.

**Affymetrix assay CNV Calling**

We used the PennCNV-Affy protocol which involves genotype calling and log 2 ratio normalized intensity calculations by Affymetrix Power Tools and SNP clustering by polar coordinates to yield b-allele frequency and log R Ratio values for each SNP for inference of CNV segments by hidden Markov model.

**CNV quality control**

We calculated Quality Control (QC) measures on our HumanHap550 GWAS data and other chip versions used based on statistical distributions to exclude poor quality DNA samples and false positive CNVs. The first threshold is the percentage of attempted SNPs which were successfully genotyped. Only samples with call rate > 98% were included. The genome wide intensity signal must have as little noise as possible. Only samples with the standard deviation (SD) of normalized intensity (LRR) < 0.35 were included. All samples were evaluated for ethnicity, based on principle components analysis. Furthermore, case and control matching were insured by calculating a genomic inflation factor between groups. Wave artifacts roughly correlating with GC content resulting from hybridization bias of low full length DNA quantity are known to interfere with accurate inference of copy number variations. Only samples where the GC corrected wave factor of LRR <|0.02| were accepted. If the count of CNV calls made by PennCNV exceeds 100, it is suggestive of poor DNA quality, and those samples were excluded. Thus, only samples with CNV call count < 100 were included. Any duplicate samples (such as monozygotic twins or repeats on the same patient) were identified and as a result one sample was excluded. The average CNV size of overlapping CNVs must meet a minimum size threshold of 1kb and not exceed a population frequency of 1%.

Trios in which both parents were processed through genotyping and met minimum quality control standards to be considered for CNV analysis. There were a total 1627 such complete trios from the CHOP CAG collected cohort genotyped on Illumina microarrays to inform de novo or inherited status of CNVs. This also adds another layer of confidence in CNV calling based on the genome-wide rates of CNV inheritance.

When CNV-disease association was evaluated via ParseCNV, red flags were reported for 13 CNV characters that could compromise resulting CNVR confidence and quality control filtering criteria has been reported in ParseCNV website (<http://parsecnv.sourceforge.net/>). We further conducted manual UCSC Track Review for spurious association. Then we further conducted manual visual review of the BAF, LRR plots of the contributing CNV calls. Next, we performed qPCR on a randomly selected set of samples carrying the CNV calls. After the above quality control steps, we achieved >90% validation success rate for detected CNVs as reported in our previous publication5.

When CNV-disease association was evaluated via ParseCNV, red flags were reported for 13 CNV characters that could compromise resulting CNVR confidence and quality control filtering criteria has been reported in ParseCNV website (http://parsecnv.sourceforge.net/)^7^

We conducted manual UCSC Track Review for spurious association. Then we further conducted manual visual review of the BAF, LRR plots of the contributing CNV calls. Next, we performed qPCR on a randomly selected set of samples carrying the CNV calls. After the above quality control steps, we achieved >90% validation success rate for detected CNVs as reported in our previous publication^7^.

**Statistical analysis of CNVs**

CNV frequency was compared between various groups, including between cases and controls. Comparisons were made for each SNP using Fisher’s exact test. To determine CNV enrichment, we only considered loci that were nominally significant between the comparative groups (*P <* 0.05). For case-control comparisons, we looked for recurrent CNVs that were observed across different independent cohorts or were not observed in any of the control subjects, and were validated with an independent method. We report statistical local minimums to narrow the association in reference to a region of nominal significance including SNPs residing within 1 Mb of each other. Resulting nominally significant CNVRs were excluded if they met any of the following criteria: i) residing on telomere or centromere proximal cytobands; ii) arising in a “peninsula” of common CNV from variation in boundary truncation of CNV calling; iii) genomic regions with extremes in GC content which produces hybridization bias; or iv) samples contributing to multiple CNVRs. Three lines of evidence establish statistical significance: independent replication *P <* 0.05, permutation of observations, and no loci observed with control enriched significance. To merge contiguous CNVRs, they must be within a short distance from each other (1MB) and of similar significance (within 1 power of ten in the negative log). These criteria were applied in this manuscript. Haplotype frequencies are estimated based on an expectation maximization (EM) algorithm implemented in Plink software. This procedure treats confidence interval maximums smaller than 0.90 as strong evidence for historical recombination.

We used DAVID (Database for Annotation, Visualization, and Integrated Discovery)^8^ to assess the significance of functional annotation clustering of independently associated results into InterPro categories. For disease-enrichment, we compared the frequency of CNVs observed in controls (n=19,584) versus that observed in cases using the Fisher’s exact test (PCNV < 5x10-8 for deletions and duplications and PHD < 9x10-4 for homozygous deletion CNVs based on a Bonferroni adjustment for genome-wide significance ‘GWS’). Chromosomal regions that aggregated multiple contiguously-significant CNVRs were subsequently merged, compared the frequency of CNVs observed in controls (n=19,584) versus that observed in cases using the Fisher’s exact test (PCNV < 5x10-8 for deletions and duplications and PHD < 9x10-4 for homozygous deletion CNVs based on a Bonferroni adjustment for genome-wide significance ‘GWS’). Chromosomal regions that aggregated multiple contiguously-significant CNVRs were subsequently merged.

**Functional enrichment analysis**

The following functional annotation types/genomic regions were considered:

1. ***Conserved***: Conserved nucleotide sequences based on PhyloP/PHASTCon^9^,
2. ***Regulatory***: CpG islands which correlate with epigenetic methylation pattern^10^, EnCODE^11^ consensus Transcription factor binding sites (TFBS), microsatellites
3. ***Coding:*** Genic or exonic region overlap based on UCSC/RefSeq^12^
4. ***Transcriptional***: experimentally-validated (miRBASE 18.0), predicted miRNA (miRNA) target sites
5. ***Known CNPs****:* established Copy Number Variation Regions (CNVRs) reported by the database of genomic variants (DGV entries)
6. ***Literature:*** Established GWAS loci based on the NHGRI GWAS catalog^13^, OMIM^14^

For each annotation category, we

1. Downloaded publically available established data
2. performed functional annotation on the identified CNVRs from this study
3. performed Monte-Carlo simulations to resample the genome for CNVRs matching equal size and type
4. performed functional annotations iteratively on these random (resampling of the genome) datasets determine whether all identified CNVRs or disease-associated CNVRs were enriched for each annotation type.

For example, for CpG islands, we downloaded the list of known mapped CpG islands from UCSC Genome Browser. We tested whether the CNVRs identified in our manuscript overlap this functional element to calculate the ‘overlap frequency’ or ‘Freq_p’. For each CNVR in a given category, e.g. Deletion CNVRs, we performed repeated sampling across the genome to simulate a set of randomly selected DNA regions of equal bp length. For each list this was done for 1,000 times. Then we tested for ‘overlap frequency’ or ‘Freq_qn’ for each dataset ‘n’.

After sampling and annotating each simulated CNVR set 1000 times, we use the permutation-derived distribution of annotation percentages for each annotation type to calculate an enrichment *P*-value such that

*P_enrich_* = 1 – ([Freq_p > Freq_qn]/1000)

We thus empirically derived the p-values based on the number of observations for the randomly generated datasets. The relative enrichment (ER) is

Freq_p/Mean(all Freq_q) across n=1:1000

This process was repeated for each of the CNVR categories (ie. Deletion CNVRs, Hom-Del CNVRs) and for each of the individual disease category-specific associated CNVRs (ie. Autoimmune/inflammatory diseases, Cardio-metabolic diseases).

**Tissue-Specific Gene Set Enrichment Analysis (TGSEA)**

With few exceptions, most genes that are known to have a causative role in autoimmune diseases have been shown to regulate molecular or subcellular processes in immune or immune-related tissues. If candidate immunologic disease-associated genes are relevant to immunologic disease biology, then expression of these genes is expected to be, on average, higher across immune or immune-related tissues (as compared to non-immune related tissues). Thus, we compared the expression of genes interrupted by CNVRs associated with autoimmune/inflammatory diseases.

We curated the expression of the transcriptome in a broad spectrum of human tissues using a publicly-available dataset consisting of summary-level, normalized gene expression levels for over 12,000 unique genes across 126 tissues and/or cell types including a large number of immune tissues/cells^15^. We downloaded the processed dataset “mean expression data matrix”.

Across the 126 unique tissues, we tested if the median or cumulative distribution of expression levels of genes impacted by CNVRs associated with autoimmune/inflammatory diseases was higher than that of the remaining transcripts in the dataset using a one-sided Wilcoxon rank (W) test or a one-sided Kolmogorov-Smirnov Test (KS), respectively. We calculated a tissue-specific gene expression “Enrichment Score” (ES) value, which is the –log_10_ (*P*-value) obtained from comparing the relative enrichment in transcript expression of genes overlapping CNVRs associated within inflammatory/autoimmune versus the transcripts of the remaining genes in the transcriptome. The above tests were done on a per tissue basis to derive a set of KS and a set of Wilcoxon ES values.

We performed the secondary immune versus non-immune comparative analysis by plotting the ES values obtained from either the Wilcoxon or KS tests by descending rank order of the respective test statistics for all 126 human tissue types. Each point represents a single tissue and is colored by whether it is classified as immune (red) or non-immune (blue), as described previously^16^**.** To formally test whether the overall ES values were higher among immune versus non-immune tissues we performed both the Wilcoxon rank sum test and the KS test on the vector of per-tissue ES values, comparing those derived from immune verses non-immune tissues.

**Supplementary Figures**

**Supplementary Figure 1**

Schematic definitions of CNVRs. The CNVR definition assigned by ParseCNV is shown as a dashed box. Rectangles represent the individual CNV call boundaries from a given sample, as provided by a CNV-calling algorithm (eg. PennCNV).

**Supplementary Figure 2**

1. Relative frequency observed of deletion or duplication CNVRs distributed by total length (in bps) affected and colored by chromosome. While there is an even distribution of lengths of CNVRs for many chromosomes, for some chromosomes (eg. Chr 5) there are sharp peaks suggesting that there is significant contribution of CNVRs of that given length. This is not observed for duplications.
2. Frequency of CNVs called across all CNVRs distributed by number of SNPs overlapping each call and the length of each call.

**Supplementary Figure 3**

1. Distribution of all CNVRs by length (in log10 bps affected) based on whether they map to a segmental duplication or recombination hotspot (feature impacted).
2. (and C) The distribution of deletion (B) and duplication (C) CNVRs by the length in bps of the CNVR and total number of samples/individuals contributing to the call, and colored by whether the CNVR overlapped one or more segmental duplications (LEFT) or recombination hotspots (RIGHT).

**Supplementary Figure 4**

1. Distribution of CNVRs that overlap recombination hotspots. Data are plotted as recombination rate by CNVR frequencies (Deletions, LEFT; Duplications, RIGHT) and data points are grouped based on population frequency.
2. Histogram of CNVRs mapping to recombination hotspots distributed by r2 (LEFT) accompanied by frequency binning of CNVRs by total length of the CNVRs by recombination rate. Data are binned based on total frequency in the population with separate plots for deletions (TOP) and duplications (BOTTOM).

**Supplementary Figure 5**

1. Distribution of all deletion (TOP) and duplication (BOTTOM) disease-associated CNVRs by length and frequency; color annotated by disease groups.
2. Distribution of all homozygous deletion (TOP) and duplication (BOTTOM) disease-associated CNVRs by length and frequency; color annotated by disease groups. A stacked bar plot is shown here allow better comparison of the contributions for a given disease type.

**Supplementary Figure 6**

1. Pleiotropic hd-CNVRs (associated with multiple disease domains) are expressed in multiple normal human tissues, consistent with the biological basis of complex multi-systemic impacts of loss of function mutations in genes mapping to these regions.
2. Enrichment for the expression of genes mapping to disease-associated CNVRs across human immune tissues as compared to non-immune tissues. Expression of all candidate genes affected by CNVRs associated with autoimmune or inflammatory diseases are evaluated across 126 human tissues/cell types. Plotted are the distribution of enrichment score (ES) values for associated genes compared to the remaining transcripts in the dataset in immune (RED) and non-immune (BLUE) cell/tissue types. Enrichment was assessed by either KS statistic (LEFT) or the Wilcoxon-rank sum test (RIGHT).

**Supplementary Figure 7**


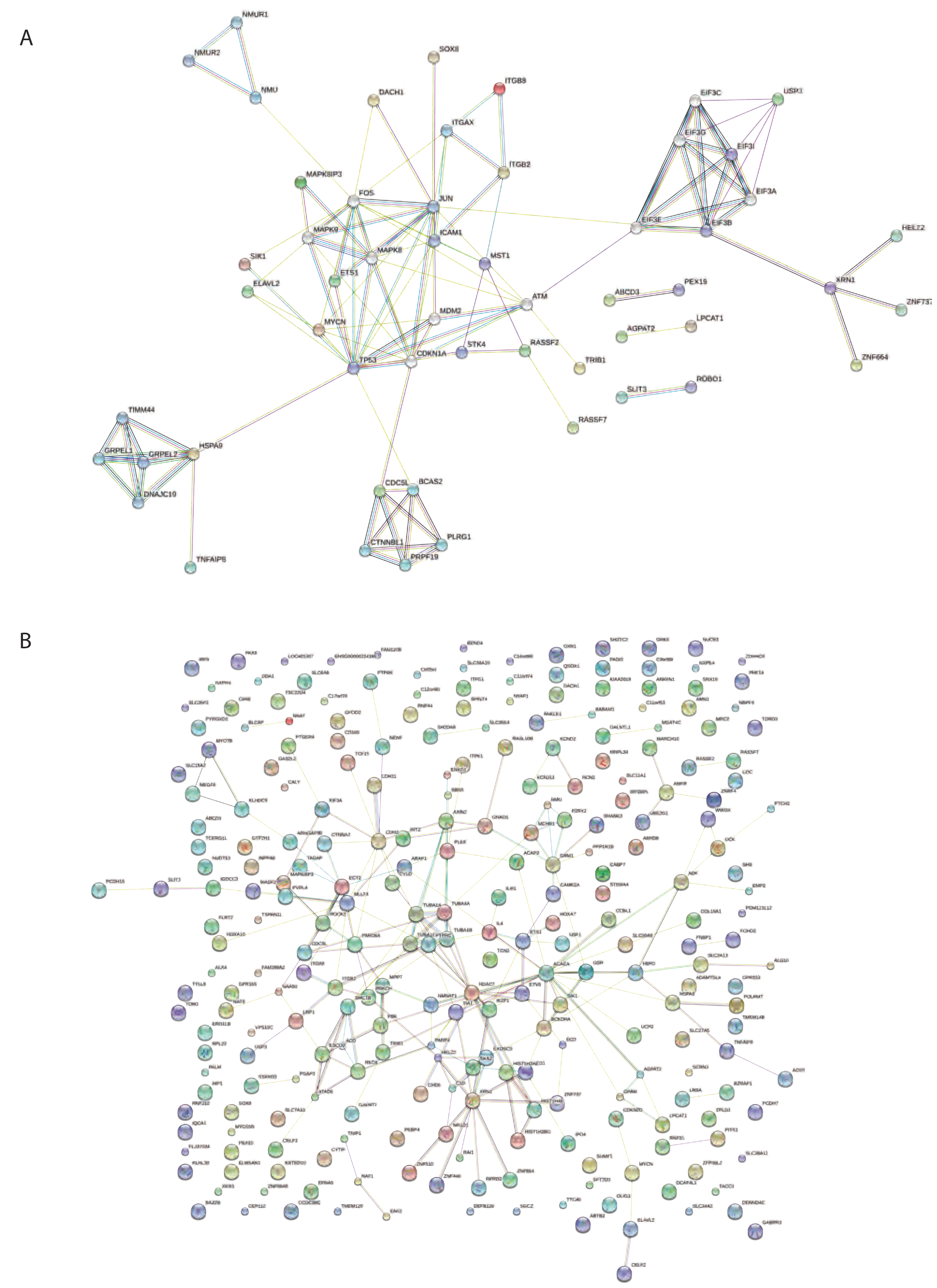


1. Example of a highly dense protein-protein interaction map identified by candidate genes affected by disease-associated CNVRs. Each dot represents one protein/gene, which is linked by the number of physical, literature reported and computationally predicted interactions.
2. In contrast, all mappable human proteins identified in the disease association analysis were included. Each dot represents one protein/gene, which is linked by the number of physical, literature reported and computationally predicted interactions. The interactions include a number of linkages between genes associated with different disease categories.

**Supplementary Figure 8**

1. Genes impacted by cancer-associated CNVRs previously reported to have a role in a wide range of solid and hematologic malignancies. Dots represent at least one prior PUBMED publication implicating a given gene as having an oncologic role in that disease site.
2. A subset of genes identified as being cancer-associated were interrogated for expression levels by RNA-seq *in silico* based on publicly available data (See methods).

**Supplementary Figure 9**

Validation of CNV Calls from Illumina Beadchip with Independent Technology qPCR. Deletions, diploid negative controls and duplications are shown. The results are sorted by qPCR level, normalized with diploid state at 1 and standard error bars are based on triplicate measurement.

**Supplementary Figure 10**

The BAF LRR Plots of the chr7p15.3 deletion in representative samples. The Log R ratio (LRR) plot and the B allele frequency (BAF) plot of the chr7p15.3 deletion in 5 representative samples are shown. The LRR plot of each sample is shown on top and the BAF plot of the same sample is shown on the bottom.

**Supplementary** **Tables**

**Supplementary Table 1.** Reference to common CNV loci (>5%) reported in the WTCCC study

| CNVR (hg18) | Length | Count SNPs | Count Del | Count Dup | RefSeq Gene | Distance from Gene | Freq | WTCCC Type | WTCCC Freq | DiffFreq | LocusID | Replicate Type |
| --- | --- | --- | --- | --- | --- | --- | --- | --- | --- | --- | --- | --- |
| chr6:31389749-31390117 | 368 | 3 | 5779 | 158 | *HLA-B* | 39511 | 0.085 | loss | 0.131 | -0.046 | 1009 | Y |
| chr8:3776007-3776955 | 948 | 3 | 5237 | 24 | *CSMD1* | 0 | 0.077 | loss | 0.0493 | 0.028 | 1012 | Y |
| chr5:117420005-117421055 | 1050 | 4 | 9688 | 4 | *DTWD2* | 779419 | 0.142 | loss | 0.0805 | 0.062 | 1001 | Y |
| chr4:10006425-10008166 | 1741 | 3 | 4974 | 11 | *ZNF518B* | 42436 | 0.073 | loss | 0.253 | -0.180 | 1014 | Y |
| chr14:21850339-21852217 | 1878 | 3 | 9823 | 60 | *DAD1* | 251430 | 0.144 | gain/loss | 0.204 | -0.060 | 5 | Y |
| chr7:52701022-52702915 | 1893 | 4 | 3 | 3462 | *POM121L12* | 367928 | 0.051 | gain | 0.0451 | 0.006 | 1024 | Y |
| chr3:65184588-65186547 | 1959 | 3 | 6671 | 0 | *MAGI1* | 128399 | 0.098 | loss | 0.0791 | 0.019 | 9 | Y |
| chr10:58186118-58188284 | 2166 | 3 | 5094 | 2 | *ZWINT* | 395078 | 0.075 | loss | 0.0492 | 0.026 | 1013 | Y |
| chr18:65360121-65362926 | 2805 | 4 | 3425 | 8 | *DOK6* | 0 | 0.050 | loss | 0.0382 | 0.012 | 1020 | Y |
| chr2:41092376-41095773 | 3397 | 5 | 10154 | 22 | *SLC8A1* | 499297 | 0.149 | loss | 0.0818 | 0.068 | 1000 | Y |
| chr3:163614102-163617940 | 3838 | 4 | 5585 | 10 | *OTOL1* | 909678 | 0.082 | loss | 0.0508 | 0.031 | 1010 | Y |
| chr13:68147928-68152545 | 4617 | 4 | 6127 | 12 | *MIR548H4* | 88799 | 0.090 | loss | 0.0596 | 0.031 | 1007 | Y |
| chr11:81183111-81188291 | 5180 | 3 | 6003 | 9 | *FAM181B* | 932403 | 0.088 | loss | 0.0819 | 0.006 | 1008 | Y |
| chr8:16306880-16312873 | 5993 | 5 | 4261 | 14 | *MSR1* | 212209 | 0.063 | loss | 0.0429 | 0.020 | 1019 | Y |
| chr3:89489946-89499754 | 9808 | 3 | 4759 | 2 | *EPHA3* | 0 | 0.070 | loss | 0.0605 | 0.009 | 1016 | Y |
| chr4:63820936-63831982 | 11046 | 3 | 4703 | 77 | *TECRL* | 994798 | 0.069 | loss | 0.084 | -0.015 | 1017 | Y |
| chr17:41544850-41562443 | 17593 | 3 | 47 | 5733 | *KIAA1267* | 0 | 0.084 | gain | 0.203 | -0.119 | 1021 | Y |
| chr6:67083141-67101257 | 18116 | 8 | 8495 | 6 | *MCART3P* | 527045 | 0.125 | loss | 0.08 | 0.045 | 1003 | Y |
| chr2:208064035-208065237 | 1202 | 4 | 7355 | 4 | *CREB1* | 37624 | 0.108 | gain | 0.179 | -0.071 | 1004 |  |
| chr8:5590045-5591685 | 1640 | 3 | 4808 | 18 | *MCPH1* | 659836 | 0.071 | gain | 0.0745 | -0.004 | 1015 |  |
| chr14:21925810-21929322 | 3512 | 3 | 4462 | 5 | *DAD1* | 174325 | 0.066 | none | none | none | 1018 |  |
| chr7:141425903-141429438 | 3535 | 4 | 7212 | 529 | *MGAM* | 0 | 0.106 | loss | NA | none | 1005 |  |
| chr1:147305744-147311729 | 5985 | 3 | 9451 | 1906 | *LOC645166* | 86066 | 0.139 | gain | 0.149 | -0.010 | 1002 |  |
| chr14:21734799-21740815 | 6016 | 3 | 3690 | 3 | *DAD1* | 362832 | 0.054 | none | none | none | 4 |  |
| chr18:64898548-64904656 | 6108 | 10 | 3900 | 164 | *CCDC102B* | 25142 | 0.057 | none | none | none | 6 |  |
| chr10:47103864-47110350 | 6486 | 3 | 1181 | 6599 | *ANTXRL* | 17890 | 0.097 | loss | 0.049 | 0.048 | 2 |  |
| chr3:53003415-53010084 | 6669 | 3 | 6675 | 4 | *SFMBT1* | 0 | 0.098 | unknown | 0.102 | -0.004 | 1006 |  |
| chr6:79063712-79070425 | 6713 | 5 | 25249 | 582 | *IRAK1BP1* | 563483 | 0.371 | gain | 0.262 | 0.109 | 11 |  |
| chr7:149081-155811 | 6730 | 3 | 39 | 3775 | *FAM20C* | 132241 | 0.056 | loss | 0.027 | 0.029 | 1022 |  |
| chr2:242583251-242593982 | 10731 | 4 | 4044 | 35 | *LOC728323* | 85535 | 0.059 | none | none | none | 7 |  |
| chr5:97090540-97102304 | 11764 | 6 | 3530 | 6 | *RIOK2* | 545779 | 0.052 | none | none | none | 10 |  |
| chr15:32530025-32543717 | 13692 | 5 | 10318 | 588 | *GOLGA8A* | 13066 | 0.152 | gain | NA | none | 999 |  |
| chr9:138619531-138638786 | 19255 | 9 | 708 | 3692 | *EGFL7* | 38412 | 0.054 | none | none | none | 1023 |  |
| chr3:75588740-75613585 | 24845 | 3 | 5270 | 23 | *FAM86D* | 21784 | 0.078 | gain | 0.0762 | 0.001 | 1011 |  |
| chr6:78384797-79088461 | 703664 | 26 | 3572 | - | *HTR1B* | 154958 | 0.053 | gain | 0.262 | -0.209 | 1026 |  |

CNVR(hg18)= CNV-region on human genome build hg18; Length= the length of the CNVR (bp); Count SNPs= the number of SNPs in each CNVR; Count Del= the number of deletion carriers; Count Dup= the number of duplication carriers; RefSeqGene=overlapping RefSeq gene; Distance from Gene= the distance between the CNVR and the RefSeq gene; Freq=the frequency of the CNVR in our study; WTCCC= Wellcome Trust Case Control Consortium; WTCCC Type=overlapping CNVs in the WTCCC study; WTCCC Freq= the frequency of the overlapping CNV in WTCCC study; DiffFreq= the difference between the frequency of the CNVR in our study and that in WTCCC study; Replicate Type=whether the CNVR found in our study was replicated by *in silico* analysis of CNVs in the WTCCC study.

**Supplementary Table 2.** Deletion CNVR without homozygous deletion CNVR overlap

| CNVR (hg18) | CountDel | RefSeqGene | Distance | DelFreq | HomDelFreq | DifDelFreqHomDelFreq |
| --- | --- | --- | --- | --- | --- | --- |
| chr5:137958990-137959777 | 684 | *HSPA9* | 19773 | 0.010055 | 0 | 0.010055 |
| chr8:126552322-126556088 | 591 | *TRIB1* | 32496 | 0.008688 | 0 | 0.008688 |
| chr12:7899399-7990569 | 533 | *SLC2A14,SLC2A3* | 0 | 0.007835 | 0 | 0.007835 |
| chr3:4071255-4076356 | 463 | *LRRN1* | 206868 | 0.006806 | 0 | 0.006806 |
| chr17:42806893-42826227 | 462 | *C17orf57* | 0 | 0.006791 | 0 | 0.006791 |
| chr6:21720928-21731694 | 460 | *SOX4* | 14100 | 0.006762 | 0 | 0.006762 |
| chr22:17257787-17388108 | 450 | *DGCR5,DGCR6,DGCR9,PRODH* | 0 | 0.006615 | 0 | 0.006615 |
| chr5:45894599-45912435 | 423 | *HCN1* | 162622 | 0.006218 | 0 | 0.006218 |
| chr3:4067922-4068619 | 411 | *LRRN1* | 203535 | 0.006042 | 0 | 0.006042 |
| chr13:59911616-59920775 | 408 | *TDRD3* | 0 | 0.005998 | 0 | 0.005998 |
| chr15:60026989-60046929 | 406 | *VPS13C* | 0 | 0.005968 | 0 | 0.005968 |
| chr9:114406899-114414974 | 396 | *KIAA1958* | 0 | 0.005821 | 0 | 0.005821 |
| chr3:4120538-4136743 | 392 | *SETMAR* | 183245 | 0.005762 | 0 | 0.005762 |
| chr3:198278255-198286437 | 389 | *DLG1* | 0 | 0.005718 | 0 | 0.005718 |
| chr3:4101259-4114899 | 372 | *SETMAR* | 205089 | 0.005468 | 0 | 0.005468 |
| chr15:60065362-60074748 | 368 | *VPS13C* | 0 | 0.00541 | 0 | 0.00541 |
| chr3:4156750-4168500 | 366 | *SETMAR* | 151488 | 0.00538 | 0 | 0.00538 |
| chr12:81692747-81730473 | 354 | *TMTC2* | 0 | 0.005204 | 0 | 0.005204 |
| chr9:114899044-114907097 | 342 | *NCRNA00256B* | 0 | 0.005027 | 0 | 0.005027 |
| chr3:198294053-198354978 | 341 | *DLG1* | 0 | 0.005013 | 0 | 0.005013 |
| chr9:28744535-28751593 | 332 | *LINGO2* | 35232 | 0.00488 | 0 | 0.00488 |
| chr3:41982343-42004839 | 330 | *ULK4* | 3679 | 0.004851 | 0 | 0.004851 |
| chr3:17494057-17513925 | 325 | *TBC1D5* | 0 | 0.004777 | 0 | 0.004777 |
| chr16:12599154-12612066 | 322 | *SNX29* | 23507 | 0.004733 | 0 | 0.004733 |
| chr14:61236316-61259284 | 321 | *HIF1A* | 0 | 0.004719 | 0 | 0.004719 |
| chr21:23788315-23809711 | 320 | *NCRNA00158* | 1870294 | 0.004704 | 0 | 0.004704 |
| chr2:76793083-76802609 | 319 | *LRRTM4* | 25757 | 0.004689 | 0 | 0.004689 |
| chr12:62269256-62405452 | 317 | *DPY19L2* | 0 | 0.00466 | 0 | 0.00466 |
| chr10:3782608-3782738 | 317 | *KLF6* | 25451 | 0.00466 | 0 | 0.00466 |
| chr3:4137607-4140733 | 312 | *SETMAR* | 179255 | 0.004586 | 0 | 0.004586 |
| chr17:30708148-30784279 | 309 | *SLFN11,SLFN12* | 0 | 0.004542 | 0 | 0.004542 |
| chr3:198426482-198448009 | 306 | *DLG1* | 0 | 0.004498 | 0 | 0.004498 |
| chr10:47129146-47173619 | 302 | *ANTXRL* | 0 | 0.004439 | 0 | 0.004439 |
| chr1:225369535-225373543 | 299 | *CDC42BPA* | 0 | 0.004395 | 0 | 0.004395 |
| chr19:19762136-19814114 | 292 | *ZNF506* | 0 | 0.004292 | 0 | 0.004292 |
| chr20:51737515-51748718 | 290 | *ZNF217* | 104472 | 0.004263 | 0 | 0.004263 |
| chr7:76068215-76395148 | 287 | *LOC100133091,POMZP3* | 0 | 0.004219 | 0 | 0.004219 |
| chr5:134370619-134375855 | 284 | *CATSPER3* | 0 | 0.004175 | 0 | 0.004175 |
| chr1:194097653-194138918 | 279 | *KCNT2* | 322618 | 0.004101 | 0 | 0.004101 |
| chr6:29454205-29454342 | 274 | *OR12D3* | 3158 | 0.004028 | 0 | 0.004028 |

CNVR(hg18)= CNV-region on human genome build hg18; CountDel= the number of deletion carriers; RefSeqGene=the closest RefSeq gene; Distance= the distance between the CNVR and the RefSeq gene; DelFreq=the frequency of the deletion in our study; HomDelFreq= the frequency of homozygous deletion; DifDelFreqHomDelFreq=the difference between the deletion frequency and the frequency of homozygous deletion.

**Supplementary Table 3. Functional Annotation Enrichment**

| category | cnvrs | mean | sim_median | sim_min | sim_max | cnvrs_enrich | p |
| --- | --- | --- | --- | --- | --- | --- | --- |
| dgvMerged | 0.99 | 0.40 | 0.40 | 0.39 | 0.42 | 2.47 | 1.00E-04 |
| cpgIslandExtUnmasked | 0.27 | 0.22 | 0.22 | 0.21 | 0.23 | 1.21 | 1.00E-04 |
| dgvMerged0 | 0.87 | 0.30 | 0.30 | 0.29 | 0.32 | 2.86 | 1.00E-04 |
| microsat | 0.32 | 0.24 | 0.24 | 0.24 | 0.25 | 1.31 | 1.00E-04 |
| omim | 0.31 | 0.11 | 0.11 | 0.09 | 0.12 | 2.94 | 1.00E-04 |
| phastConsElements44way | 0.62 | 0.49 | 0.49 | 0.48 | 0.50 | 1.28 | 1.00E-04 |
| phyloP44wayAll | 0.99 | 0.75 | 0.75 | 0.74 | 0.76 | 1.32 | 1.00E-04 |
| rdmr | 0.12 | 0.08 | 0.08 | 0.07 | 0.08 | 1.61 | 1.00E-04 |
| targetScanS | 0.18 | 0.12 | 0.12 | 0.11 | 0.13 | 1.45 | 1.00E-04 |
| tfbsConsSites | 0.56 | 0.44 | 0.44 | 0.43 | 0.45 | 1.27 | 1.00E-04 |
| wgRna | 0.04 | 0.03 | 0.03 | 0.02 | 0.03 | 1.44 | 1.00E-04 |
| genic | 0.59 | 0.40 | 0.40 | 0.38 | 0.41 | 1.48 | 1.00E-04 |
| gwasCatalog | 0.25 | 0.16 | 0.16 | 0.16 | 0.17 | 1.52 | 1.00E-04 |
| exonic | 0.33 | 0.23 | 0.23 | 0.22 | 0.24 | 1.44 | 1.00E-04 |

category=the annotation category for CNVRs; cnvrs=the percentage of CNVRs in our study in each annotation category; mean=the mean percentage of sets of randomly distributed CNVRs simulated across the genome in each annotation category; sim_median= the median percentage of sets of randomly distributed CNVRs simulated across the genome in each annotation category; sim_min= the minimal percentage of sets of randomly distributed CNVRs simulated across the genome in each annotation category; sim_max= the maximal percentage of sets of randomly distributed CNVRs simulated across the genome in each annotation category; cnvrs_enrich=the fold of enrichment of CVNRs in our study compared to that of simulated CNV sets.

**Supplementary Table 4: Disease Categories Present in Cohort by Genotyping Platform Version**

| Cohort | Version | Samples | Probes | Aid/Aif | Cancer | Cardio-metabolic | Healthy | Neuro |
| --- | --- | --- | --- | --- | --- | --- | --- | --- |
| CHOP CAG | BDCHP-1X10-HUMANHAP550 | 8,213 | 555,352 SNPs | 2,746 | 1,046 | 612 | 3,062 | 520 |
| CHOP CAG | HumanHap550v3 | 11,010 | 561,466 SNPs | 884 | 478 | 1,089 | 6,192 | 1,610 |
| CHOP CAG | HumanHap550-2_v3-1 | 12,601 | 561,466 SNPs | 3,054 | 1,810 | 0 | 4,706 | 2,257 |
| CHOP CAG | Human610-Quadv1 | 28,582 | 620,901 (592,532 SNPs + 28,369 CN) | 4,805 | 5,771 | 880 | 10,073 | 2,750 |
| CHOP CAG | Human660W-Quad_v1 | 5,442 | 657,366 (561,490 SNPs + 95,876 CN) | 0 | 0 | 0 | 0 | 5,440 |
| CHOP CAG | Human1M-Duov3-0 | 2,180 | 1,199,187 (1,154,691 SNPs + 44,496 CN) | 0 | 0 | 0 | 0 | 2,179 |
| UW | SignatureChip PN v2.0 12-plex | 169 | 340,000 | 0 | 0 | 0 | 0 | 169 |
| UW | Agilent NICHD 44 | 367 | 430,000 | 0 | 0 | 0 | 0 | 367 |
| UW | SignatureChip PN v1.1 12-plex | 863 | 540,000 | 0 | 0 | 0 | 0 | 863 |
| UW | SignatureChip OS v1.1 | 4,296 | 970,000 | 0 | 0 | 0 | 0 | 4,296 |
| UW | SignatureChip OS v1.1 Rev. B | 4,557 | 970,000 | 0 | 0 | 0 | 0 | 4,557 |
| UW | SignatureChip OS v1.0 | 314 | 1,040,000 | 0 | 0 | 0 | 0 | 314 |
| UW | SignatureChip OS v2.0 12-plex | 16,754 | 1,350,000 | 0 | 0 | 0 | 0 | 16,754 |
| UW | SignatureChip OS v3.0 12-plex | 1,765 | 1,370,000 | 0 | 0 | 0 | 0 | 1,765 |
| HGDP | HumanHap650Yv3_A | 984 | 650,000 | 0 | 0 | 0 | 984 | 0 |
| NINDS (Coriell 550K) | HumanHap550v3_A | 441 | 550,000 | 0 | 0 | 0 | 441 | 0 |
| NINDS (317K+240K) | Illumina317K+240K | 227 | 557,000 | 0 | 0 | 0 | 227 | 0 |
| PARC (CAP and PRINCE) | Illumina317K | 936 | 317,000 | 0 | 0 | 0 | 936 | 0 |
| London (Parents) | Illumina550K | 760 | 550,000 | 0 | 0 | 0 | 760 | 0 |
| PARC2 (CAP2) | Human610-Quadv1_B | 232 | 610,000 | 0 | 0 | 0 | 232 | 0 |
| PARC2 (PRINCE2) | Illumina610KQuad | 534 | 610,000 | 0 | 0 | 0 | 534 | 0 |
| FHCRC | Human610-Quadv1_B | 1,430 | 610,000 | 0 | 0 | 0 | 1,430 | 0 |
| inChianti | HumanHap550v3_a | 695 | 550,000 | 0 | 0 | 0 | 695 | 0 |
| WTCCC2(NBS) | CustomIllumina1.2M | 2,090 | 1,200,000 | 0 | 0 | 0 | 2,090 | 0 |
| ARIC | AffymetrixSNP6 | 8,733 | 2,000,000 | 0 | 0 | 0 | 8,733 | 0 |
| WTCCC2(58C) | AffymetrixSNP6 | 2,523 | 2,000,000 | 0 | 0 | 0 | 2,523 | 0 |

Cohort= the cohort in our study; version= the chip type that each cohort was genotyped on; samples=the number of samples in each cohort; probes= the number of probes on the chip type that each cohort was genotyped on; Aid/Aif= the number of samples with autoimmune disorders or autoinflammatory diseases in each cohort; cancer= the number of samples with cancer diagnosis in each cohort; Cardio-metabolic= the number of samples with cardio-metabolic disorders in each cohort ; Healthy= the number of healthy individuals in each cohort; Neuro= the number of samples with neuropsychiatric disorders in each cohort.

The CNV study cohort is composed of 68,028 samples in the CHOP CAG cohort genotyped on the Illumina array, 29,085 samples in the UW case cohort genotyped on array CGH, and 19,585 samples in the UW healthy control genotyped on Illumina and Affymetrix array.

**References**

1. Shaikh, T. H. *et al.* High-resolution mapping and analysis of copy number variations in the human genome: a data resource for clinical and research applications. *Genome Res.* **19,** 1682–90 (2009).

2. Craddock, N. *et al.* Genome-wide association study of CNVs in 16,000 cases of eight common diseases and 3,000 shared controls. *Nature* **464,** 713–20 (2010).

3. Wang, K. *et al.* PennCNV: an integrated hidden Markov model designed for high-resolution copy number variation detection in whole-genome SNP genotyping data. *Genome Res.* **17,** 1665–74 (2007).

4. Colella, S. *et al.* QuantiSNP: an Objective Bayes Hidden-Markov Model to detect and accurately map copy number variation using SNP genotyping data. *Nucleic Acids Res.* **35,** 2013–2025 (2007).

5. Diskin, S. J. *et al.* Adjustment of genomic waves in signal intensities from whole-genome SNP genotyping platforms. *Nucleic Acids Res.* **36,** e126 (2008).

6. Glessner, J. T. & Hakonarson, H. Genome-wide association: from confounded to confident. *Neuroscientist* **17,** 174–84 (2011).

7. Glessner, J. T., Li, J. & Hakonarson, H. ParseCNV integrative copy number variation association software with quality tracking. *Nucleic Acids Res.* **41,** e64 (2013).

8. Dennis, G. *et al.* DAVID: Database for Annotation, Visualization, and Integrated Discovery. *Genome Biol.* **4,** P3 (2003).

9. Nguyen, D.-Q. *et al.* Reduced purifying selection prevails over positive selection in human copy number variant evolution. *Genome Res.* **18,** 1711–1723 (2008).

10. Bird, A. P. CpG-rich islands and the function of DNA methylation. *Nature* **321,** 209–13

11. Boyle, A. P. *et al.* Annotation of functional variation in personal genomes using RegulomeDB. *Genome Res.* **22,** 1790–7 (2012).

12. TH, P. The human genome browser at UCSC. *Genome Res.* **12,** 996 (2002).

13. NHGRI. Published GWAS through 08/01/2014. *NHGRI GWA Catalog* (2014).

14. Hamosh, A., Scott, A. F., Amberger, J. S., Bocchini, C. A. & McKusick, V. A. Online Mendelian Inheritance in Man (OMIM), a knowledgebase of human genes and genetic disorders. *Nucleic Acids Res.* **33,** D514-7 (2005).

15. Benita, Y. *et al.* Gene enrichment profiles reveal T-cell development, differentiation, and lineage-specific transcription factors including ZBTB25 as a novel NF-AT repressor. *Blood* **115,** 5376–84 (2010).

16. D, T. *et al.* Proteins encoded in genomic regions associated with immune-mediated disease physically interact and suggest underlying biology. *PLoS Genet.* **7,** e1001273 (2011).
