# Supplementary material for: Rare copy number variants in over 100,000 European ancestry subjects reveal multiple disease associations
Source: Nat Commun. 2020 Jan 14;11:255. doi: 10.1038/s41467-019-13624-1 (PMC6959272; doi:10.1038/s41467-019-13624-1)
Supplement: Supplementary file 9 — Description of Additional Supplementary Files [file 41467_2019_13624_MOESM9_ESM.pdf]

Title: Supplementary Data 1.

Description: Deletion Frequency Optimized CNVRs (provided in Excel format)

Title: Supplementary Data 2.

Description: Duplication Frequency Optimized CNVRs (provided in Excel format)

Title: Supplementary Data 3.

Description: Homozygous Deletion Frequency Optimized CNVRs (provided in Excel format)

Title: Supplementary Data 4.

Description: Poor Linkage Disequilibrium Between Rare CNV and Common SNP Genotypes (provided in Excel format)

Title: Supplementary Data 5.

Description: Extended results showing CNVRs associated with diseases reaching GWS significance (5x10-8). (provided in Excel format)

Title: Supplementary Data 6.

Description: Extended results showing hdCNVRs associated with diseases reaching experimentally defined significance based on repeated simulations (5x10-4). (provided in Excel format)

Title: Supplementary Data 7.

Description: Publications supporting somatic impact of cancer-associated CNVR mapped genes in multiple human cancers (provided in Excel format)
